# RELATIONSHIP BETWEEN MEDIA IMAGE, PERCEIVED AGE STEREOTYPE, AND AGING ANXIETY IN KOREAN OLDER ADULTS

**DOI:** 10.1093/geroni/igad104.2152

**Published:** 2023-12-21

**Authors:** Miri Kim, Soondool Chung

**Affiliations:** Ewha Womans University, Seoul, Republic of Korea; Ewha Womans University, Seoul, Republic of Korea

## Abstract

This study aimed to investigate the relationship between media image, perceived age stereotype, and aging anxiety in older adults in South Korea, in order to gain a better understanding of the psychosocial and financial-related anxieties faced by older adults in later life. Given that South Korea is projected to become the most aged nation in the world within 50 years, studying aging anxiety in this context is particularly important. A total of 600 older adults from 65 to 84 years of age were analysed using structural equation modelling and bootstrapping method, using a cross-sectional, secondary data from OO Research Institute of South Korea. The findings indicated that media image was positively correlated with perceived age stereotype (β=.380,p<.001), aging anxiety on financial factors (β=.145,p<.001), and aging anxiety on psychosocial factors (β=.136,p<.01), while perceived age stereotype was correlated with aging anxiety on psychosocial factors (β=.205,p<.001). In terms of indirect effects, perceived age stereotype mediated the relationship between media image and aging anxiety on psychosocial factors (95% CI: 0.034 to 0.121). The results suggest that older adults who encounter more negative images of old age and aging in the media are more likely to develop perceptions of age stereotype, which in turn increases their aging anxiety on psychosocial factors. Practical implementations are suggested to reduce aging anxiety. The study’s findings have implications for reducing aging anxiety and understanding the experiences of older adults in the era of population aging.

